# Pressmen and Weekly Contributions

**Published:** 1920-11-20

**Authors:** 


### Pressmen and Weekly Contributions.

THE *Western Mail* announces that every member of the staff is joining in the scheme for a weekly contribution to King Edward VII. Hospital, Cardiff. This participation is part of an effort to extend the system of weekly contributions in the city.

